# Exploring the association of disease-modifying therapies for multiple sclerosis and BTK inhibitors with epilepsy

**DOI:** 10.1177/17562864241276204

**Published:** 2024-09-21

**Authors:** Afsaneh Shirani, Nil Saez-Calveras, Jack P. Antel, Moein Yaqubi, Wayne Moore, Amy L. Brewster, Olaf Stuve

**Affiliations:** Department of Neurological Sciences, University of Nebraska Medical Center, Omaha, NE, USA; Department of Neurology, University of Texas Southwestern Medical Center, Dallas, TX, USA; Parkland Memorial Hospital, Dallas, TX, USA; Department of Neurology and Neurosurgery, Montreal Neurological Institute, McGill University, Montreal, QC, Canada; Department of Neuroscience, Montreal Neurological Institute, McGill University, Montreal, QC, Canada; Department of Pathology and Laboratory Medicine, and International Collaboration on Repair Discoveries (ICORD), University of British Columbia, Vancouver, BC, Canada; Department of Biological Sciences, Southern Methodist University, Dallas, TX, USA; Department of Neurology, University of Texas Southwestern Medical Center, 5323 Harry Hines Blvd., Dallas, TX 75390-9096, USA; Neurology Section, VA North Texas Health Care System, Dallas, TX, USA; Peter O’Donnell Jr. Brain Institute, University of Texas Southwestern Medical Center, Dallas, TX, USA

**Keywords:** BTK inhibitors, disease-modifying therapies, epilepsy, FAERS database, multiple sclerosis

## Abstract

**Background:**

Multiple lines of evidence suggest a role of inflammation in epilepsy. Seizure incidence in patients with multiple sclerosis (MS) is twofold to threefold higher than the age-matched general population.

**Objectives:**

To explore the association of MS disease-modifying therapies (DMTs) and FDA-approved Bruton tyrosine kinase inhibitors (for lymphocytic malignancies) with the occurrence of epilepsy using the US Food and Drug Administration Adverse Event Reporting System (FAERS) database.

**Design:**

Secondary analysis of the FAERS database.

**Methods:**

We conducted a disproportionality analysis of FAERS between 2003-Q4 and 2023-Q3. MS DMTs and the Bruton tyrosine kinase inhibitor, ibrutinib, were included in the analysis. An inverse association was defined by a 95% confidence interval (CI) upper limit of reporting odds ratio (ROR) <1.

**Results:**

We found an inverse association of ibrutinib, ocrelizumab, ofatumumab, rituximab, and teriflunomide with epilepsy. The strongest inverse association was seen with ibrutinib (ROR: 0.338; 95% CI: 0.218–0.524).

**Conclusion:**

Our findings suggest the possibility of considering these medications for repurposing opportunities in epilepsy and support a potential pathogenic role of leukocyte subsets in seizure perpetuation.

## Plain language summary

How multiple sclerosis treatments and Bruton tyrosine kinase inhibitors may be linked to epilepsy

The research presented in this manuscript attempts to elucidate the potential relationship between inflammation and epilepsy. Multiple sclerosis (MS) is the most common inflammatory disorder of the brain and spinal cord in humans. There are over 20 FDA-approved disease-modifying therapies (DMTs) for MS. Other anti-inflammatory agents called Bruton tyrosine kinase (BTK) inhibitors are in clinical development. We show that the use of some DMTs and a BTK inhibitor appear to be associated with a lower chance of epilepsy.

## Introduction

Epilepsy is a neurologic disorder characterized by recurrent seizures affecting over 70 million people worldwide.^1^ It is well established that neuroinflammation occurs in association with epileptic seizures and may contribute to the progression of this disorder. In recent years, histological and transcriptomic evidence further demonstrated altered inflammatory pathways and microglial reactivity in epilepsy. Analysis of human refractory epilepsy brain tissue has revealed increased complement activation and aberrant microglial phagocytic signaling.^2^ The levels of immune inhibitory molecules CD47, its receptor signal-regulatory protein α, and CD200 were also downregulated in resected brain tissue from pediatric patients with medically intractable epilepsy.^3^ Experimental models of both genetic and acquired epilepsies recapitulate the findings in the epileptic human brain.^4–9^ Furthermore, peripheral blood myeloid cells have been shown to invade the central nervous system (CNS) during or after a seizure, which may exacerbate inflammation.^5,10^ For example, C-C motif chemokine receptor type 2-positive (CCR2^+^) monocytes populated the hippocampus of mice 1–3 days after an event of status epilepticus (SE). Meanwhile, CCR2-deficiency (CCR2^–/–^) decreased SE-induced monocyte CNS recruitment and reduced the levels of the proinflammatory cytokine interleukin-1β (IL-1β) in the hippocampus. These animals also had accelerated weight regain, reduced blood–brain barrier (BBB) disruption, and attenuated neuronal damage.^5^

Additional indirect evidence favoring a potential role of the immune system in the pathogenesis of epilepsy comes from epidemiological studies in multiple sclerosis (MS), the most prevalent chronic inflammatory disorder of the CNS in humans. The incidence of seizures in patients with MS is twofold to threefold above the general age-matched population,^11^ and MS represents a risk factor for refractory epilepsy. Cortical inflammation and demyelinating lesions may lead to the formation of epileptogenic neuronal foci in these patients.^12^ Proinflammatory cytokines IL-1β, interleukin-6, and tumor necrosis factor-α secreted by glial cells are elevated in the CNS of both patients with MS^13^ and epilepsy^14^ and can influence neuronal excitability.^14^ Interestingly, generalized seizures can manifest during the clinical presentation at disease onset.^15^ This implies that seizure activity can, in turn, contribute to MS worsening, potentially by inducing the expression of adhesion molecules in the endothelium, loss of BBB integrity, and promoting the transmigration of immune cells, thus increasing local inflammation. These observations suggest that epileptic seizures may perpetuate inflammation in persons with MS and that an inflammatory CNS environment in MS may trigger and perpetuate seizures.

There are currently no approved anti-inflammatory pharmaceutical agents for patients with epileptic seizures. However, immunomodulatory agents are now considered a part of the standard of care for encephalitis-associated seizures and certain forms of refractory epilepsy. In MS, there are currently 23 FDA-approved disease-modifying therapies (DMTs). All these DMTs reduce the numbers of circulating leukocyte subsets, sequester them out of the CNS, or modulate their differentiation to be less inflammatory. CNS-penetrant Bruton tyrosine kinase (BTK) inhibitors—a new class of medications capable of targeting both B cells and myeloid cells—are currently being evaluated in clinical trials for their potential use in MS. At the time of this publication, no BTK inhibitors are FDA approved for MS.

In this study, we aimed to explore the association of DMTs for MS and FDA-approved BTK inhibitors (for indications other than MS) with the occurrence of epilepsy using reports from the US Food and Drug Administration Adverse Event Reporting System (FAERS). Pharmacovigilance data from FAERS, primarily utilized for post-marketing safety signal detection by screening for disproportionate reporting, can additionally serve as a data mining resource for identifying inverse associations and potential opportunities for medication repurposing.^16^

## Methods

FAERS is a publicly available database that houses data on spontaneous reporting of adverse events (AEs) and medication errors submitted to the FDA. We conducted a disproportionality analysis of FAERS spanning from the fourth quarter of 2003 to the third quarter of 2023 using the OpenVigil2.1-MedDRA-v24 software^17^ to identify associations of MS DMTs as well as FDA-approved BTK inhibitors (for lymphocytic malignancies) with epilepsy. Disproportionality analysis is a validated method for detecting significant associations between drugs and AEs. The DMTs for MS investigated in our study comprised “interferon-β-1b,” “interferon-β-1a,” “glatiramer acetate,” “teriflunomide,” “dimethyl fumarate,” “fingolimod,” “cladribine,” “natalizumab,” “alemtuzumab,” “rituximab,” “ofatumumab,” “ocrelizumab,” and “ublituximab.” We also investigated FDA-approved BTK inhibitors including “ibrutinib,” “acalabrutinib,” “zanubrutinib,” and “pirtobrutinib.” BTK inhibitors are currently in clinical trial programs for potential use in the treatment of MS. As there are currently no FDA-approved BTK inhibitors for MS, and the FAERS database exclusively includes FDA-approved products, we focused on FDA-approved BTK inhibitors used in treating lymphocytic malignancies (such as chronic lymphocytic leukemia and mantle cell lymphoma) which were the only FDA-approved BTK inhibitors available during our study period.

For AE of interest, we used the preferred term “epilepsy.” To ensure the inclusion of reports where the drugs of interest were listed as concomitant medications, rather than exclusively as primary or secondary suspects for the AE of interest, we applied no filter based on the role of the drug. Drugs associated with ⩽5 drug-event pairs (i.e., drug–epilepsy combinations) were excluded from the analysis.

Disproportionality was primarily quantified using the reporting odds ratio (ROR) and its 95% confidence interval (CI). ROR represents the odds of a certain AE (in this case, “epilepsy”) occurring with the drug of interest, compared to the odds of the same AE occurring with all other drugs in the database (Supplemental Table 1). An inverse association was defined when the upper limit of the 95% CI for ROR was <1. A safety signal (AE signal) was defined based on Evans criteria^18^ which requires the following: ⩾3 reports, proportional reporting ratio (PRR) ⩾2, and χ^2^ value ⩾4. PRR is the proportion of reports for a certain event occurring with the drug of interest divided by the corresponding proportion for all other drugs in the database (Supplemental Table 1).

## Results

**Figure 1.**
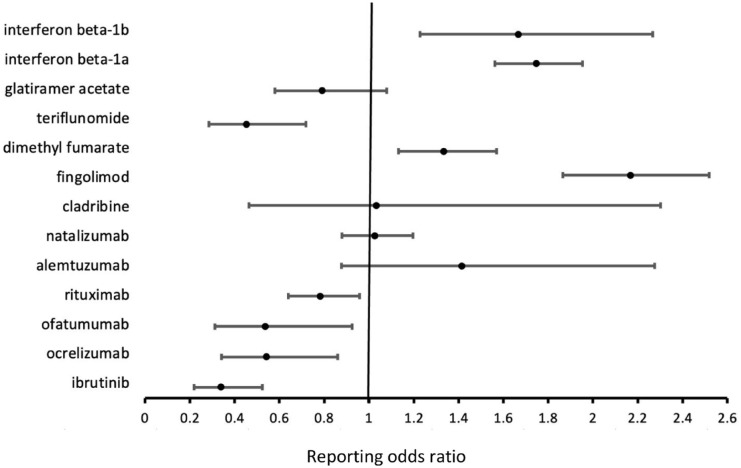
Disproportionality analysis demonstrating the association between epilepsy and various immunotherapies based on the data from the FDA Adverse Event Reporting System database. The reporting odds ratio indicates the odds of a certain adverse event (in this case, “epilepsy”) occurring with the drug of interest, compared to the odds of the same adverse event occurring with all other drugs in the database. Drugs associated with fewer than five drug-event (drug–epilepsy) pairs, including acalabrutinib, zanubrutinib, and ublituximab, as well as those with no existing reports in the database, including pirtobrutinib, were excluded from the disproportionality analysis.

Table 1 shows the number of reports of epilepsy as an AE versus reports of AEs other than epilepsy for drugs of interest versus all other drugs, based on the FAERS database. The following medications were not included in the disproportionality analysis due to an insufficient number of reports or lack of any reports: acalabrutinib, zanubrutinib, pirtobrutinib, and ublituximab. The results of disproportionality analysis are displayed in Figure 1. Inverse associations were found between the following drugs and epilepsy: ibrutinib (ROR: 0.338; 95% CI: 0.218–0.524), ocrelizumab (ROR: 0.541; 95% CI: 0.341–0.859), ofatumumab (ROR: 0.536; 95% CI: 0.311–0.924), rituximab (ROR: 0.782; 95% CI: 0.639–0.957), and teriflunomide (ROR: 0.452; 95% CI: 0.285–0.718). A safety signal for epilepsy was found for fingolimod based on the Evans criteria (PRR: 2.164; 95% CI: 1.863–2.513, χ^2^ = 106.06).

**Table 1.** Number of reports of epilepsy as an adverse event versus reports of adverse events other than epilepsy for drugs of interest versus all other drugs based on the data from the FDA Adverse Event Reporting System database.

Drug of interest	Mechanism of action	No. of reports of epilepsy associated with the drug of interest	No. of reports of events other than epilepsy associated with the drug of interest	No. of reports of epilepsy associated with all other drugs	No. of reports of events other than epilepsy associated with all other drugs
Ibrutinib	BTK inhibitor	20	45,349	15,253	11,676,511
Acalabrutinib	BTK inhibitor	1	4157	15,272	11,717,703
Zanubrutinib	BTK inhibitor	3	970	15,270	11,720,890
Pirtobrutinib^a^	BTK inhibitor	n/a	n/a	n/a	n/a
Ocrelizumab	Anti-CD20 humanized mAb	18	25,512	15,255	11,696,348
Ofatumumab	Anti-CD20 fully human mAb	13	18,586	15,260	11,703,274
Ublituximab	Anti-CD20 chimeric mAb	0	41	15,273	11,721,819
Rituximab	Anti-CD20 chimeric mAb	95	93,024	15,178	11,628,836
Alemtuzumab	Anti-CD52 humanized mAb	17	9233	15,256	11,712,627
Natalizumab	Anti-α4-integrin humanized mAb	164	122,778	15,109	11,599,082
Cladribine	Adenosine deaminase-resistant analog of deoxyadenosine; disrupting DNA synthesis in B and T lymphocytes	6	4458	15,267	11,717,402
Fingolimod	S1PR modulator	173	61,642	15,100	11,660,218
Dimethyl fumarate	Activation of NrF2 transcriptional pathway	144	83,136	15,129	11,638,724
Teriflunomide	Blocking de novo pyrimidine synthesis	18	30,504	15,255	11,691,356
Glatiramer acetate	Modulating T-cell differentiation from pro-inflammatory Th1 to anti-inflammatory Th2	40	38,818	15,233	11,683,042
Interferon beta-1a	Modulating secretion of pro and anti-inflammatory cytokines, suppressing T-cell activation	319	141,438	14,954	11,580,422
Interferon beta-1b	Modulating secretion of pro and anti-inflammatory cytokines, suppressing T-cell activation	41	18,897	15,232	11,702,963

a As of the third quarter of 2023, no records were available for pirtobrutinib in the FAERS database since it received FDA approval in January 2023.

BTK, Bruton tyrosine kinase; FAERS, US Food and Drug Administration Adverse Event Reporting System; mAb, monoclonal antibody; NrF2, nuclear factor erythroid 2-related factor 2; S1PR, sphingosine-1-phosphate receptor.

## Discussion

Our results suggest that the FDA-approved BTK inhibitor ibrutinib, as well as the anti-CD20 monoclonal antibodies including ocrelizumab, ofatumumab, and rituximab, are associated with reduced odds of epilepsy. In addition, the dihydro-orotate dehydrogenase (DHODH) inhibitor, teriflunomide showed a similar inverse association. Among other DMTs, fingolimod met the criteria for safety signal detection for epilepsy based on the Evans criteria (⩾3 reports, PRR ⩾2, and χ^2^ value ⩾4). Our observations are interesting, as they suggest that agents that reduce or modulate B lymphocytes (ibrutinib and the anti-CD20 agents) or reduce the proliferation of activated T and B lymphocytes (teriflunomide) may be inversely associated with epileptic seizures. The strongest inverse association was seen with ibrutinib, which has also been shown to modulate myeloid cell subsets, including CNS microglia.^19^ Our results appear to be in line with the findings of a recent systematic review and network meta-analysis of seizure risk in patients with MS treated with DMTs that demonstrated a trend toward a lower risk ratio with rituximab (anti-CD20).^20^

BTK inhibitors have emerged as a new family of potential therapeutic agents for patients with MS.^19^ These oral agents, including ibrutinib, were initially proposed as B-cell targeting agents for the treatment of lymphocytic malignancies. However, increasing evidence suggests that BTK inhibitors can also modulate myeloid cell function. BTK mediates Fc gamma-receptor III intracellular signaling, regulating inflammasome activation, reactive oxygen species production, and cytokine secretion.^21^ In these cells, BTK also interacts with MyD88 and TIR-domain-containing adapter molecule 1 to facilitate Toll-like receptor (TLR) signaling leading to nuclear factor-kappa B activation and interferon-β production.^22^ BTK also mediates the upregulation of TLR expression by intracellular major histocompatibility complex class II and CD40 molecules. In addition, postmortem studies of human MS brains have demonstrated increased BTK expression in MS lesion samples,^23^ and the use of CNS-penetrating BTK inhibitors in MS clinical trials have shown some promise in phase II trials. Several phase III study programs are currently ongoing. It is this immune modulatory activity against myeloid cells, coupled with their ability to penetrate the CNS that could potentially explain the inverse association observed with ibrutinib in our study.

In addition to the possible involvement of myeloid cells and microglia, studies have also evidenced the role of the adaptive immune response in some forms of epilepsy. It is well known that cytotoxic T-cell infiltrates and autoantibodies produced by B cells are associated with certain seizure-related disorders, including autoimmune and paraneoplastic encephalitis, viral encephalitis, and Rassmussen’s encephalitis. In this context, the anti-CD20 agent, rituximab, constitutes a treatment option for patients with new-onset refractory SE and febrile infection-related epilepsy syndrome. In addition, a systematic review identified that the use of anti-CD20 agents could be beneficial in patients with refractory SE and drug-resistant epilepsy.^24^ The findings of our study, in which anti-CD20 agents (rituximab, ocrelizumab, and ofatumumab) were all associated with decreased odds of epilepsy, also seem to support a potential beneficial effect of these agents in epilepsy.

Our observation on the effects of teriflunomide is consistent with recent findings on its impact on mitochondrial DHODH, a regulator of neuronal activity in hippocampal networks, namely, teriflunomide is an inhibitor of DHODH that has been shown to suppress mean firing rates via synaptic and intrinsic excitability, and that ameliorated susceptibility to seizures in the Dravet syndrome epilepsy model.^25^

In contrast with the above, we found that fingolimod appeared to be associated with an increased odds of having epilepsy. This is in line with a recent meta-analysis of the incidence of seizures in individuals with MS participating in randomized clinical trials of DMTs, which found that sphingosine-1-phosphate receptor (S1PR) modulators, including fingolimod, were associated with more than a twofold increase in the risk of seizures when compared with placebo or comparators.^26^ Given the wide distribution of S1PRs in the CNS, it remains to be explained whether the effect of S1PR modulators on neuronal excitability may explain this observation.

It is important to point out the limitations of the FAERS database. The database does not definitely establish a causal (or inverse) relationship between product exposure and reported events. The absence of a control group complicates distinguishing between associations and coincidences. Moreover, relying on voluntary reporting means that FAERS may miss certain AEs, and the accuracy of the reported AEs may not be verifiable. The database’s nature precludes adjustment for clinical characteristics that could have impacted the use of certain drugs and reliable evaluation of synergistic effects. It also remains possible that not all the submitted reports might have been based on the same definition of epilepsy. In addition, the lack of a known denominator (i.e., the total number of prescriptions for the product of interest) precludes the calculation of incidence rates using the FAERS data. Finally, caution needs to be taken regarding the generalizability of the results about the potential inverse association between ibrutinib and epilepsy in patients with MS, given ibrutinib is used for lymphocytic malignancies, and there is yet no FDA-approved BTK inhibitor for MS.

## Conclusion

In summary, given the known mechanisms of action of BTK inhibitors, anti-CD 20 monoclonal antibody, and teriflunomide, our findings hint at the possibility of considering these medications for potential repurposing opportunities for the treatment of epilepsy and provide plausibility for a potential pathogenic role of leukocyte subsets in the perpetuation of epileptic seizures.

## Supplemental Material

sj-docx-1-tan-10.1177_17562864241276204 – Supplemental material for Exploring the association of disease-modifying therapies for multiple sclerosis and BTK inhibitors with epilepsy Supplemental material, sj-docx-1-tan-10.1177_17562864241276204 for Exploring the association of disease-modifying therapies for multiple sclerosis and BTK inhibitors with epilepsy by Afsaneh Shirani, Nil Saez-Calveras, Jack P. Antel, Moein Yaqubi, Wayne Moore, Amy L. Brewster and Olaf Stuve in Therapeutic Advances in Neurological Disorders

## Declarations

**Ethics approval and consent to participate:** Not applicable since this study was based on publicly available anonymous data from the FDA Adverse Event Reporting System database.

**Consent for publication:** Not applicable since this was a brief report presenting the secondary analysis of a large dataset.

**Competing interests:** A.S. serves on the editorial board of *Multiple Sclerosis and Related Disorders*, *Journal of Central Nervous System Disease, and Brain Sciences*. She is also an associate editor for *Frontiers in Neurology* and *Frontiers in Immunology* and an editor for the Multiple Sclerosis section of *Current Treatment Options in Neurology*. She has received an honorarium for serving on the advisory medical board for TG therapeutics. O.S. serves on the editorial boards of *Therapeutic Advances in Neurological Disorders*, *Expert Review of Clinical Immunology*, and he is a section editor for *Current Treatment Options in Neurology*, has served on data monitoring committees for Genentech-Roche, and Novartis without monetary compensation, has advised EMD Serono, Novartis, and Octave Bioscience, receives grant support from EMD Serono, is a 2021 recipient of a Grant for Multiple Sclerosis Innovation (GMSI), Merck KGaA, is funded by a Merit Review grant (federal award document number (FAIN) BX005664-01) from the United States (U.S.) Department of Veterans Affairs, Biomedical Laboratory Research and Development, is funded by RFA-2203-39314 (PI) and RFA-2203-39305 (co-PI) grants from the National Multiple Sclerosis Society (NMSS). O.S. is an Associate Editor of *Therapeutic Advances in Neurological Disorders*; therefore, the peer review process was managed by alternative members of the Board and the submitting Editor was not involved in the decision-making process. A.L.B., J.P.A., N.S.-C., M.Y., and W.M. have nothing to disclose.

**Availability of data and materials:** Study data are publicly available through the FDA Adverse Event Reporting System database.
