# Supplementary material for: Exploring the association of disease-modifying therapies for multiple sclerosis and BTK inhibitors with epilepsy
Source: Ther Adv Neurol Disord. 2024 Sep 21;17:17562864241276204. doi: 10.1177/17562864241276204 (PMC11456174; doi:10.1177/17562864241276204)
Supplement: sj-docx-1-tan-10.1177_17562864241276204 – Supplemental material for Exploring the association of disease-modifying therapies for multiple sclerosis and BTK inhibitors with epilepsy [file sj-docx-1-tan-10.1177_17562864241276204.docx]

**Supplementary Table 1.** Contingency table for disproportionality analysis

|  | Drug of interest | All other drugs | Total |
| --- | --- | --- | --- |
| Adverse event of interest | a | b | a+b |
| All other adverse events | c | d | c+d |
| Total | a+c | b+d | a+b+c+d |

In the context of a 2x2 contingency table for disproportionality analysis, 'a' represents the number of reports for the adverse event of interest associated with the drug of interest, 'b' is the number of reports for the adverse event of interest associated with all other drugs, 'c' represents the number of reports for all other adverse events associated with the drug of interest, and 'd' is the number of reports for all other adverse events associated with all other drugs. The formulas used for calculation of chi square value (χ2), reporting odds ratio (ROR), and proportional reporting ratio (PRR) are shown below.

χ2 = $\frac{\left( ad-bc \right)^{2} (a+b+c+d)}{\left( a+b \right)(c+d)(a+c)(b+d)}$ ROR = $\frac{\frac{a}{c}}{\frac{b}{d}}$ PRR = $\frac{\frac{a}{a+c}}{\frac{b}{b+d}}$
